# Phenotypic Variability Among Patients With D4Z4 Reduced Allele Facioscapulohumeral Muscular Dystrophy

**DOI:** 10.1001/jamanetworkopen.2020.4040

**Published:** 2020-05-01

**Authors:** Lucia Ruggiero, Fabiano Mele, Fiore Manganelli, Dario Bruzzese, Giulia Ricci, Liliana Vercelli, Monica Govi, Antonio Vallarola, Silvia Tripodi, Luisa Villa, Antonio Di Muzio, Marina Scarlato, Elisabetta Bucci, Giovanni Antonini, Lorenzo Maggi, Carmelo Rodolico, Giuliano Tomelleri, Massimiliano Filosto, Stefano Previtali, Corrado Angelini, Angela Berardinelli, Elena Pegoraro, Maurizio Moggio, Tiziana Mongini, Gabriele Siciliano, Lucio Santoro, Rossella Tupler

**Affiliations:** 1 Department of Neurosciences, Reproductive, and Odontostomatological Sciences, University Federico II, Naples, Italy; 2 Department of Life Sciences, University of Modena and Reggio Emilia, Modena, Italy; 3 Department of Preventive Medical Sciences, Federico II University, Naples, Italy; 4 Neurological Clinic, Department of Clinical and Experimental Medicine, University of Pisa, Italy; 5 Center for Neuromuscular Diseases, Department of Neurosciences, University of Turin, Turin, Italy; 6 Department of Neurosciences, University of Padua, Padua, Italy; 7 Neuromuscular Unit, Fondazione IRCCS Ca’Granda Ospedale Maggiore Policlinico, Dino Ferrari Center, University of Milan, Milan, Italy; 8 Center for Neuromuscular Disease, Center for Excellence on Aging, Gabrile D’Annunzio University Foundation, Chieti, Italy; 9 Neuromuscular Repair Unit, Inspe and Division of Neuroscience, IRCSS San Raffaele Scientific Institute, Milan, Italy; 10 Department of Neuroscience, Mental Health, and Sensory Organs, S. Andrea Hospital, University of Rome Sapienza, Rome, Italy; 11 IRCCS Foundation, C. Besta Neurological Institute, Milan, Italy; 12 Department of Clinical and Experimental Medicine, University of Messina, Messina, Italy; 13 Neurology Clinic, Spedali Civili Hospital, Brescia, Italy; 14 IRCCS San Camillo, Venezia, Italy; 15 Child Neurology and Psychiatry Unit, IRCCS, Casimiro Mondino Foundation, Pavia, Italy; 16 Department of Molecular, Cell, and Cancer Biology, University of Massachusetts Medical School, Worcester; 17 Li Weibo Institute for Rare Diseases Research at the University of Massachusetts Medical School, Worcester

## Abstract

**Importance:**

Facioscapulohumeral muscular dystrophy (FSHD) is considered an autosomal dominant disorder, associated with the deletion of tandemly arrayed D4Z4 repetitive elements. The extensive use of molecular analysis of the D4Z4 locus for FSHD diagnosis has revealed wide clinical variability, suggesting that subgroups of patients exist among carriers of the D4Z4 reduced allele (DRA).

**Objective:**

To investigate the clinical expression of FSHD in the genetic subgroup of carriers of a DRA with 7 to 8 repeat units (RUs).

**Design, Setting, and Participants:**

This multicenter cross-sectional study included 422 carriers of DRA with 7 to 8 RUs (187 unrelated probands and 235 relatives) from a consecutive sample of 280 probands and 306 relatives from the Italian National Registry for FSHD collected between 2008 and 2016. Participants were evaluated by the Italian Clinical Network for FSHD, and all clinical and molecular data were collected in the Italian National Registry for FSHD database. Data analysis was conducted from January 2017 to June 2018.

**Main Outcomes and Measures:**

The phenotypic classification of probands and relatives was obtained by applying the Comprehensive Clinical Evaluation Form which classifies patients in the 4 following categories: (1) participants presenting facial and scapular girdle muscle weakness typical of FSHD (category A, subcategories A1-A3), (2) participants with muscle weakness limited to scapular girdle or facial muscles (category B, subcategories B1 and B2), (3) asymptomatic or healthy participants (category C, subcategories C1 and C2), and (4) participants with myopathic phenotypes presenting clinical features not consistent with FSHD canonical phenotype (category D, subcategories D1 and D2).

**Results:**

A total of 187 probands (mean [SD] age at last neurological examination, 53.5 [15.2] years; 103 [55.1%] men) and 235 relatives (mean [SD] age at last neurologic examination, 45.1 [17.0] years; 104 [44.7%] men) with a DRA with 7 to 8 RUs and a molecular diagnosis of FSHD were evaluated. Of 187 probands, 99 (52.9%; 95% CI, 45.7%-60.1%) displayed the classic FSHD phenotype, whereas 86 (47.1%; 95% CI, 39.8%-54.3%) presented incomplete or atypical phenotypes. Of 235 carrier relatives from 106 unrelated families, 124 (52.8%; 95% CI, 46.4%-59.7%) had no motor impairment, whereas a small number (38 [16.2%; 95% CI, 9.8%-23.1%]) displayed the classic FSHD phenotype, and 73 (31.0%; 95% CI, 24.7%-38.0%) presented with incomplete or atypical phenotypes. In 37 of 106 families (34.9%; 95% CI, 25.9%-44.8%), the proband was the only participant presenting with a myopathic phenotype, while only 20 families (18.9%; 95% CI, 11.9%-27.6%) had a member with autosomal dominant FSHD.

**Conclusions and Relevance:**

This study found large phenotypic variability associated with individuals carrying a DRA with 7 to 8 RUs, in contrast to the indication that a positive molecular test is the only determining aspect for FSHD diagnosis. These findings suggest that carriers of a DRA with 7 to 8 RUs constitute a genetic subgroup different from classic FSHD. Based on these results, it is recommended that clinicians use the Comprehensive Clinical Evaluation Form for clinical classification and, whenever possible, study the extended family to provide the most adequate clinical management and genetic counseling.

## Key Points

**Question:**

What are the phenotypes expressed among patients with facioscapulohumeral muscular dystrophy (FHSD) who are carriers of D4Z4 reduced allele with 7 to 8 repeat units?

**Findings:**

In this cross-sectional study of 187 probands and 235 relatives who carry a D4Z4 reduced allele with 7 to 8 repeat units, 47.1% of probands did not have the classic FSHD phenotype, and 52.8% of the carrier relatives were nonpenetrant. In 106 families, 18.9% had a member with autosomal dominant FSHD, whereas in 34.9%, the proband was the only participant expressing a myopathic phenotype.

**Meaning:**

The findings of this study suggest that knowledge of phenotypic variation in the expression of D4Z4 reduced allele with 7 to 8 repeat units in individuals with FSHD could be informative for clinical management and genetic counseling.

## Teaser

This cross-sectional study investigates the clinical expression of facioscapulohumeral muscular dystrophy (FSHD) in the genetic subgroup of carriers of D4Z4 reduced allele with 7 to 8 repeat units.

## Introduction

Facioscapulohumeral muscular dystrophy (FSHD; OMIM 158900) is among the most common forms of hereditary myopathy.^1^ At present, 2 genetically distinct disease subtypes, FSHD1 and FSHD2, are described^2,3^ on the basis of molecular features. In FSHD1, representative of 95% of patients, the molecular variation resides in a stretch of tandemly arrayed 3.3-kb repetitive elements named D4Z4. Patients with FSHD1 carry D4Z4 alleles with 10 or fewer repeat units (RUs), with autosomal dominant inheritance.^4^ In FSHD2, individuals carry 2 D4Z4 arrays in the healthy range (ie, >10 RUs), but approximately 80% of these patients have a mutation in the *SMCHD1* gene (OMIM 614982) with D4Z4 reduced CpG methylation and a permissive 4qA haplotype. It has to be noted that *SMCHD1* variants as well as D4Z4 hypomethylation in the presence of the haplotype 4qA/PAS distal to the D4Z4 array have been found in patients with bosma arhinia and microphtalmia syndrome, a congenital disease with no associated muscle phenotype.^5,6,7,8,9,10,11,12^

The classic FSHD phenotype is characterized by onset in the first or second decade of life with progressive facial, shoulder girdle, and pectoral muscle weakness and atrophy, often asymmetric.^13^ Disease progression may lead to the involvement of abdominal muscles and distal lower extremity weakness, causing a steppage gait before impairment of pelvic girdle muscles.^14^

Patients with the smallest number of RUs display more severe phenotypes, including earlier wheelchair use and increased frequency of extramuscular manifestations.^15^ In contrast, patients with the largest number of residual D4Z4 fragments (ie, 7-10 RUs) have a milder disease and no affected relatives.^16^

However, since its discovery, molecular analysis of the D4Z4 locus for FSHD diagnosis has revealed an unanticipated complexity, without a straightforward association of the clinical phenotype with molecular variations.^17^ Furthermore, the autosomal dominant mode of inheritance has come into question because there are families in which the disease appears only in 1 generation or in a single individual.^18,19,20,21,22,23^ Several reports describe atypical phenotypes in carriers of D4Z4 reduced alleles (DRAs).^24^ In some of these cases, additional investigations revealed the presence of variants in neuromuscular disorder genes that can explain the atypical clinicaphenotypes.^25,26,27,28,29,30,31,32,33,34,35,36,37,38,39,40,41^ Moreover, D4Z4 alleles in the range of FSHD1 (ie, 4-8 RUs) are carried by 3% of the healthy population.^5,19,20^ We also found that 1.3% of healthy people carry 1 DRA associated with the permissive haplotype 1614qA, and 2% carry 1 DRA with the 4qA allele.^5^ These observations argue for the role of modifying loci or epigenetic mechanisms influencing the clinical expression of disease.^42^

In the present study, we applied the Comprehensive Clinical Evaluation Form (CCEF), a clinical tool developed to systematically describe clinical phenotypes in individuals with suspected FSHD, with the aim of obtaining additional information about the clinical significance of detecting a DRA with 7 to 8 RUs. These alleles have a 2 in 10 frequency (eFigure 1 in the Supplement) in the population accrued in the Italian National Registry for FSHD (INRF) and 1.7% in the general population^5^; therefore, their detection has high clinical relevance but requires additional knowledge to establish their value for diagnosis and genetic counseling. At present, a standardized genotype-phenotype correlation analysis of probands and relatives does not exist for carriers of a DRA with 7 to 8 RUs.^43,44,45,46,47^ Here we evaluate whether this genetic subgroup is different from those with classic FSHD.

## Methods

### Study Design and Participants

We performed a cross-sectional study of 187 probands (ie, the family member who first manifested symptoms and was the first individual analyzed) and 235 relatives from a consecutive group of 280 probands and 306 relatives, all carriers of a DRA with 7 to 8 RUs, accrued by the INRF between 2008 and 2016. All participants included in this study carry 1 DRA associated with the permissive haplotype 4qA. We did not analyze the short sequence-length polymorphism in all participants, given that several studies have shown that different haplotypes can be carried by patients with FSHD.^5,6,7,8,9,10,11,12^ The carriers of DRA with 7 to 8 RUs represent 20% of all carriers accrued by INRF (eFigure 1 in the Supplement). We enrolled only patients for whom clinical evaluation was performed with the CCEF by a properly trained physician who belonged to the Italian Clinical Network for FSHD (ICNF). The ICNF is distributed across Italy and includes 1 diagnostic laboratory and 14 clinical centers. All clinical and molecular data were collected in the INRF database. Participant recruitment was approved by the Ethics Committee of Modena and all participating centers. Written informed consent, according to the Declaration of Helsinki, was obtained from each participant enrolled in the study. This study followed the Strengthening the Reporting of Observational Studies in Epidemiology (STROBE) reporting guideline for cross-sectional studies.

### Procedures

#### Clinical Investigation

We applied the CCEF, a recently published^48^ novel clinical standardized clinical tool with interrater reliability.^48^ The CCEF consists of 4 sections. The first section, the evaluation form, investigates the patient’s clinical history and disability and assesses muscle segmental involvement. The second section includes the FSHD evaluation scale to calculate the FSHD score (range, 0-15).^49^ The combination of the clinical features summarized in the clinical diagnostic form (section 3) assigns patients to different phenotypic categories (section 4). Participants presenting with facial and scapular girdle muscle weakness typical of FSHD are classified as category A, subcategories A1 to A3; those with muscle weakness limited to scapular girdle or facial muscles are assigned to category B, subcategories B1 and B2, respectively; those who are asymptomatic or healthy are assigned to category C, subcategories C1 and C2; and those with myopathic phenotypes presenting clinical features not consistent with the FSHD canonical phenotype are assigned to category D, subcategories D1 and D2.

#### Molecular Characterization

As previously described,^15^ allele sizes were estimated by Southern hybridization with probe p13E-11 of 7 μg of *Eco*RI-digested, *Eco*RI/BlnI-digested genomic DNA extracted from peripheral blood lymphocytes, electrophoresed in a 0.4% agarose gel, for 45 to 48 hours at 35 V, alongside an 8- to 48-kb marker (BioRad). Participants carrying DRA with 7 to 8 RUs were included in the study. To distinguish between DRAs from chromosome 10q and 4q, DNA from each proband was analyzed by NotI digestion and hybridization with the B31 probe to confirm the chromosome 4q origin of the 33- to 35-kb *Eco*RI allele. Restriction fragments were detected by autoradiography or by using the Typhoon Trio system (GE Health).

### Statistical Analysis

Numerical variables were described using mean (SD) and compared between groups using 1-way analysis of variance, followed by Tukey honest significance post hoc test, in cases of more than 2 groups, or the *t* test for independent samples, in cases of 2-group comparisons. Linear models were used to adjust comparisons with respect to potential confounding factors. Categorical variables were synthetized using absolute frequencies with percentages, and differences in distribution were assessed using the χ^2^ test. We obtained 95% CIs on proportions using the exact method for binomial and multinomial proportions. Missing values were not imputed. All reported *P* values were 2-sided, and statistical significance was set at a *P* < .05. All statistical analyses were performed with R version 3.5.0 (R Project for Statistical Computing).

## Results

We reevaluated 187 unrelated probands (mean [SD] age at last neurological examination, 53.5 [15.2] years). In the 103 men (55.1%), the mean (SD) age was 49.9 (15.5) years; in the 84 women (44.9%), it was 57.9 (13.6) years (*P* < .001). We identified 235 related carriers of DRA with 7 to 8 RUs from 106 unrelated families. The number of relatives tested in each family was variable, with a range of 1 to 10 members, with a mean (SD) of 2.2 (1.6) relatives for each family. Among family members, mean (SD) age at last neurological examination was 45.1 (17.0) years; in the 104 men (44.7%), it was 39.4 (15.1) years, and in the 131 women (55.3%), it was 49.5 (17.2) years (*P* < .001) (Table).

**Table.** Clinical Summary of Probands and Relatives

Characteristic	Probands				Relatives			
	All (n = 187)	Men (n = 103)	Women (n = 84)	*P* value	All (n = 235)	Men (n = 104)	Women (n = 131)	*P* value
Age at evaluation, mean (SD), y	53.5 (15.2)	49.9 (15.5)	57.9 (13.6)	<.001	45.1 (17.0)	39.4 (15.1)	49.5 (17.2)	<.001
Age at onset, mean (SD), y	33.3 (17.9)	28.8 (16.2)	39.1 (18.3)	<.001	33.4 (17.3)^a^	25.7 (12.3)^a^	38.8 (18.4)^a^	<.001
FSHD score, mean (SD), y	5.8 (3.4)	5.7 (3.5)	6.0 (3.2)	.66	3.6 (3.0)^a^	3.2 (2.6)^a^	3.9 (3.2)^a^	.25

Abbreviation: FSHD, facioscapulohumeral muscular dystrophy.

^a^ Calculated with 107 relatives (46 men; 61 women) with FSHD symptoms.

### Distribution of Clinical Categories Among Probands

**Figure 1.**
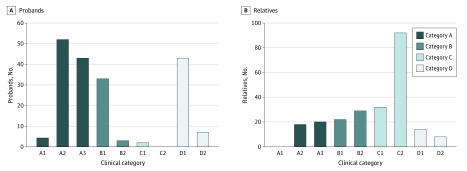
Description of Clinical Phenotypes Observed Among Probands and Relatives Category A includes patients with typical facioscapulohumeral muscular dystrophy, presenting facial and scapular girdle muscle weakness without atypical features. Patients with this typical phenotype are further subdivided in 3 subcategories (A1-A3). Category B includes patients with muscle weakness limited to scapular girdle (B1) or facial (B2) muscles. Category C includes asymptomatic individuals without motor impairment. This group is further divided in 2 subcategories, as follows: C1, patient with minor signs; and C2, patients with completely normal results from a neurologic examination. Category D comprises atypical phenotypes. In particular, those assessed as category D1 present some facioscapulohumeral muscular dystrophy features with other uncommon characteristics suggestive of the possible copresence of an additional muscle disease. Patients in category D2 do not fulfil the diagnostic criteria for facioscapulohumeral muscular dystrophy.

**Figure 2.**
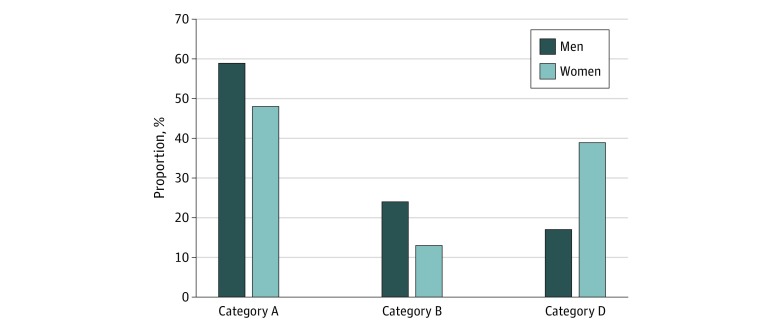
Distribution of Sex Across Clinical Category

We grouped the probands in clinical categories on the basis of their clinical phenotype (Figure 1A): 99 (52.9%; 95% CI, 45.7%-60.1%) displayed the classic FSHD phenotype, and were classified as category A, whereas 86 (47.1%; 95% CI, 39.8%-54.3%) presented incomplete or atypical phenotypes, with 36 (19.3%; 95% CI, 14.0%-25.8%) displaying incomplete FSHD phenotype (category B), 33 (17.6%; 95% CI, 12.6%-24.0%) presenting shoulder involvement without facial weakness (category B1), and 50 (26.7%; 95% CI, 20.7%-33.8%) with atypical clinical features not consistent with classic FSHD (category D). We observed a significantly different distribution of gender across clinical categories (*P* = .002) (Figure 2). In particular, the number of men (24 [72.7%; 95% CI, 54.2%-86.1%]) showing a facial-sparing phenotype (category B1) was higher compared with women (9 [27.3%; 95% CI, 13.9%-45.8%]), whereas the atypical phenotypes (category D) were more frequent in women than men (33 [66.0%; 95% CI, 51.1%-78.4%] vs 17 [34.0%; 21.6%-48.9%]). Age at last clinical evaluation was significantly different among categories with patients in category D being older (mean [SD] age, 59.0 [13.7] years) than those with classic (mean [SD] age, 52.2 [15.1] years; *P* = .007) or incomplete (mean [SD] age, 50.1 [15.7] years; *P* = .009; *P *for analysis of variance = .01) FSHD phenotypes.

### Distribution of Clinical Categories Among Relatives

Clinical evaluation of 235 relatives (Figure 1B) showed that 38 (16.2%; 95% CI, 9.8%-23.1%) displayed the classic FSHD phenotype (category A), and 124 (52.8%; 95% CI, 46.4%-59.7%) had no muscle weakness (category C). A total of 73 relatives (31.0%; 95% CI, 24.7%-38.0%) presented with incomplete or atypical phenotypes, with 51 (21.7%; 95% CI, 16.7%-27.6%) with an incomplete FSHD phenotype (category B), 14 (5.9%; 95% CI, 3.4%-10.0%) with uncommon characteristics suggestive of a possible comorbidity (category D1), and 8 (3.4%; 95% CI, 1.6%-6.8%) with clinical features not consistent with FSHD (category D2). No differences were observed across categories of relatives with respect to sex (*P* = .09).

### Distribution of Clinical Categories in Families

**Figure 3.**
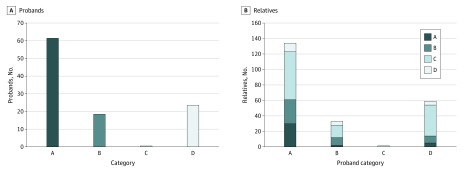
Distribution of Clinical Categories Of Relatives in Relationship With the Clinical Category of Probands Only probands with at least 1 family member available for the analysis (106 of 187 [56.7%]) were included.

Facioscapulohumeral muscular dystrophy is characterized by great variability in clinical expression. To investigate this aspect, we grouped families based on the proband’s clinical category and subgrouped them based on the clinical patterns assessed in relatives (Figure 3; eFigure 2 in the Supplement). Among all families with the proband assessed as category A, we found that 30 relatives (22.4%; 95% CI, 15.8%-30.6%) were assessed as category A. Only in 10.0% (95% CI, 4.1%-21.2%) of families (6 of 60) did all relatives display the classic FSHD phenotype. In contrast, 39 families (65.0%; 95% CI, 51.5%-76.6%) had at least 1 nonpenetrant carrier, and in 19 families (31.7%; 95% CI, 20.6%-45.1%), all relatives carrying 1 DRA were nonpenetrant. When we considered families with the proband classified as category B, 10 relatives (30.3%; 95% CI, 16/2%-48.9%) were assessed as category B, whereas 16 (48.5%; 95% CI, 31.2%-66.1%) were nonpenetrant carriers. Considering families with the proband classified as category D, 40 relatives (67.8%; 95% CI, 54.2%-79.0%) were nonpenetrant carriers (eTable 1 in the Supplement). Finally, in one-third of families (37 [34.9%; 95% CI, 25.9%-44.8%]), the proband was the only participant who presented a myopathic phenotype, while only 20 families (18.9%; 95% CI, 11.9%-27.0%) had a member with an autosomal dominant FSHD. The percentages of relatives with classic FSHD phenotype in families with the proband classified as category B and D were 6.1% (2 relatives) and 8.5% (5 relatives), respectively.

### Age at Onset

The mean (SD) age at onset estimated among probands was 33.3 (17.9) years, and 124 (66.3%) presented with first symptoms when older than 20 years. Mean (SD) age at onset was significantly different between men and women (28.8 [16.2] vs 39.1 [18.3]; *P* < .001) (eTable 2 in the Supplement). Age at onset of probands was also different across categories, with participants with classic FSHD phenotypes (ie, category A) having significantly earlier onset than those with atypical phenotypes (ie, category D) (mean [SD] age, 29.1 [16.4] vs 40.9 [17.8] years; *P* = .001; *P *for analysis of variance < .001). We found the same pattern of difference in mean (SD) age of onset in relatives (men vs women: 25.7 [12.3] years vs 38.8 [18.4] years; *P* = .001; category A vs D: 29.2 [17.6] years vs 43.6 [18.0] years; *P* = .02). The mean (SD) age at onset of symptomatic relatives (33.4 [17.3] years) was lower than the mean (SD) age at last neurological examination of relatives with no muscle weakness (ie, category C; 41.1 [15.3] years) (eTable 2 in the Supplement).

At onset, 178 of 246 patients (72.4%) reported scapular girdle weakness, 13 of 246 (5.3%) referred to signs of facial weakness, and 31 (12.2%) presented with pelvic girdle weakness. In the latter group, 24 of 31 (77.4%) were women.

### Severity of Motor Impairment

**Figure 4.**
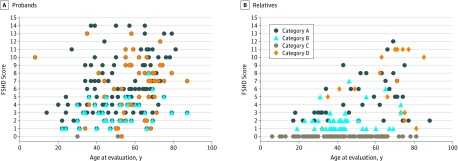
Severity of Muscle Impairment In Probands and Relatives Degree of motor impairment, measured as facioscapulohumeral muscular dystrophy score, is described on the basis of age at last clinical evaluation and clinical category in probands (A) and relatives (B).

We also established the degree of motor impairment among probands using the FSHD clinical score (Figure 4). The mean (SD) FSHD score was 5.8 (3.4). We did not detect a significant difference in FSHD score between men and women (5.7 [3.5] vs 6.0 [3.2]; *P* = .66). Instead, we detected a difference in FSHD score among probands and symptomatic relatives, with the latter presenting less severe clinical impairment (mean [SD] FSHD score, 5.8 [3.4] vs 3.6 [3.6]; *P* < .001).

Our analysis also revealed that participants assessed as category A developed a more severe disease than subjects assessed as category B (mean [SD] FSHD score, 6.7 [3.3] vs 3.1 [1.6]; *P* < .001). This difference remained significant even after adjusting the comparison by age at onset and disease duration (difference in FSHD score, 3.2; 95% CI, 2.1-4.3; *P* < .001). We found no significant difference in FSHD score between participants classified as category A and those classified as category D, even though the muscles affected in the 2 subgroups are different. In particular, facial weakness significantly contributed to the whole FSHD score in subjects assessed as category A compared with those in category D (mean [SD] contribution, 25.0% [12.8%] vs 16.4% [20.0%]; *P* = .006), whereas limb girdle muscles significantly contributed to the whole FSHD score in those assessed as category D compared with those assessed as category A (mean [SD] contribution of impairment of limb girdle muscles: 29.4% [17.9%] vs 13.2% [14.9%]; *P* < .001). This estimate was achieved evaluating the contribution of each subscore to the whole score.

## Discussion

In FSHD genotype-phenotype correlation studies, the idea that there is an inverse correlation between the number of D4Z4 repeats and the severity of the disease has been favored.^43^ Alleles with DRA with 1 to 3 RUs were generally associated with a more severe form of disease, while DRA with 4 to 8 RUs was associated with the classic form of FSHD.^15,16^ However, it is now clear that many different phenotypes can be observed among individuals carrying a DRA, even DRAs of the same size, with critical consequences for clinical management.^24^

The recently published evidence-based guidelines for the diagnosis and management of FSHD^50^ proposed that molecular testing, including the measurement of the size of D4Z4 alleles, the presence of 4qA polymorphism, and the D4Z4 methylation status, become a determinant aspect for diagnosis, whereas clinical features are not taken into account. In this study, we showed that among carriers with DRA with 7 to 8 RUs, the molecular test was not sufficient for diagnosis. Considering the phenotypic variability of the probands and the high percentage of nonpenetrant individuals among relatives, finding a D4Z4 contraction might have little diagnostic and prognostic significance. We suggest applying the CCEF as a tool for the standard evaluation of the phenotype in conjunction with the molecular test. Our study showed that it is mandatory to extend the molecular test to the largest number of family members for proper genetic counselling.

Our analysis of 422 participants also provided elements for managing diagnosis, prognosis, and counseling among carriers of DRA with 7 to 8 RUs. We showed that this molecular group constitutes a very heterogeneous clinical group, including phenotypes different from the classic form of FSHD. We observed that 52.9% of probands had the classic FSHD phenotype (ie, category A in the CCEF), whereas the rest (47.1%) displayed incomplete or atypical phenotypes. Among 187 probands, very few (4.2%) had severe facial involvement (category A1). This is a peculiar clinical aspect; in fact, we have shown previously that the percentage of patients with a classical phenotype in the carriers of smaller DRAs was close to 80% among carriers of DRA with 1 to 3 RUs.^15^ In addition, the percentage of relatives who are asymptomatic carriers was lower, ranging from 9.5% (for DRA with 1-3RUs) to 27.6% (for DRA with 4-6 RUs).^18^ Instead, the phenotypic expression of probands and relatives who carry 1 DRA with 7 to 8 RUs is quite similar to those with 9 to 10 RUs (G. Ricci, PhD, unpublished data, 2020). Therefore, the group with DRA with 7 to 8 RUs is not a classic FSHD group, as suggested by the FSHD guidelines. Instead, the characteristics are more similar to those found in carriers of borderline alleles. At onset, most participants (71.9%) reported scapular girdle weakness, and 17.6% presented with a facial-sparing phenotype (ie, category B1). Thus, in patients with a DRA with 7 to 8 RUs, facial involvement was less frequent and less severe than previously reported among individuals carrying DRA with fewer RUs.^15^ In our cohort, most symptomatic patients reported the first symptom when they were older than 20 years, without a statistically significant difference between the mean of age at onset for probands and relatives. Therefore, we can consider carriers of a DRA with 7 to 8 RUs as late-onset patients.^51^

This observation is also very interesting from another point of view. In our cohort, we reported several families with relatives in 3 generations, and the absence of statistically significant difference between the mean age at onset for probands and relatives suggests that no anticipation was detectable in our sample.

Among probands, 26.7% displayed atypical signs (ie, category D) and showed some distinctive features. First, patients in category D reported disease onset at older than 40 years. Therefore, we can consider them late-onset patients. Second, no probands assigned to category D displayed autosomal dominant inheritance; rather, they were sporadic cases. In this subgroup, most patients presented with some FSHD features as well as other uncommon characteristics suggestive of the possible copresence of an additional disease (ie, subcategory D1). In addition, 3.7% of these patients presented no signs that met the diagnostic criteria for FSHD (ie, subcategory D2). Considering that 3% of general population^5^ carry a DRA with 4 to 8 RUs, some patients in category D2 have a different myopathy, and the association with the DRA with 7 to 8 RUs might be attributed to random occurrence. To our knowledge, this was the first study in which a large group of myopathic carriers of the molecular defect associated with FSHD1 was identified. These patients did not meet the clinical criteria, and they indicate alternative diagnoses. The next step is to perform muscle biopsies and exome sequencing to identify other causative genes in this subgroup of patients.

The evaluation of the FSHD clinical score confirms that myopathic carriers of 1 DRA with 7 to 8 RUs had a mild clinical impairment,^16,18,52^ but at the same time, we observed large variability of clinical expression, particularly among probands. Family studies show that this variability does not depend exclusively on disease duration. We also found that most relatives (52.8%) carrying a DRA with 7 to 8 RUs had no muscle weakness. This percentage is much higher than the 25% to 30% reported by other studies.^18,23,52^ This difference is not because of the age at last clinical evaluation, given that asymptomatic and nonpenetrant relatives in our cohort were older than the mean age at onset of symptomatic relatives. Thus, it is likely that they will never develop disease.

In 34.9% of families, all relatives were healthy, irrespective of the proband’s clinical category. This observation shows that disease penetrance varied among families and indicates that the genetic background or the presence of comorbidities might modulate disease onset and development. Our data point at the possibility that, in the heterozygous state, a D4Z4 reduction might produce a subclinical, sensitized condition that requires another contributing factor to cause overt myopathy. In some cases, it might be the simultaneous heterozygosity for a different and recessive myopathy, as suggested by many reports, in which the FSHD contractions are found in association with a second molecular variation.^25,26,27,28,29,30,31,32,33,34,35,36,37,38,39,40,41^ This possibility is also consistent with previous reports of expression changes of candidate proteins that were associated with FSHD in some families but were unchanged when other families were examined. It is also plausible that drugs or toxic agents might contribute to disease onset and clinical variability. In this respect, anamnestic records documented by the CCEF may provide useful information. Consequently, an extended evaluation of the family context is necessary to estimate prognosis for patients carrying or at risk of carrying DRA with 7 to 8 RUs.

Finally, by evaluating age at onset in combination with FSHD score and clinical category, we found that women had a later onset and frequently display atypical phenotypes. It is commonly reported that women have a milder phenotype, but the reasons are not well known.^53^ Our data suggest the role of sex-specific factors that delay disease onset in women or accelerate or facilitate disease appearance in men. Considering the mean age at onset in woman, we can hypothesize a crucial role of hormonal factors related to fertile age, but this hypothesis should be confirmed by dedicated studies. It is also possible that factors expressed by men (eg, testosterone is a potent anabolic factor promoting muscle protein synthesis and muscular regeneration) create a major sensitivity to the alterations caused by the FSHD pathogenic mechanism among men.^54^ Moreover, men and women may respond differently to catabolic conditions because of their hormonal profiles.^55,56^

### Limitations

This study has some limitations. The CCEF is an extensive clinical tool, which takes about 20 minutes to apply. Only a physician with expertise in neuromuscular disease can use the tool correctly. Thus, it is preferable they be properly trained. Second, a long follow-up period may be necessary to evaluate whether some symptomatic patients will be assigned to a different clinical category or if some nonpenetrant relatives will develop any sign of muscle impairment.

Third, most nonpenetrant relatives were older than 20 years, and the mean (SD) of age at last neurological examination (ie, 41.1 [15.3] years) was older than that of symptomatic relatives (ie, 33.4 [17.3] years). Thus, it is likely that they will never develop disease or that they might develop some symptoms at older age. In our cohort we had several patients with atypical phenotypes who developed the disease when older than 40 years. The clinical follow-up of nonpenetrant subjects will provide relevant clinical information on this matter.

## Conclusions

The findings of this study indicate that in the case of probands who carry a DRA with 7 to 8 RUs and do not present the classic FSHD phenotype, it is necessary to consider alternative myopathies. In sporadic cases presenting with atypical phenotypes, the random association of a myopathic phenotype with a contracted D4Z4 allele has to be considered, given that there is a 1.7% frequency of DRA with 7 to 8 RUs in the general population. This study showed that the genetic background can influence the penetrance and phenotypic expression of disease in relatives carrying the same molecular signature. Based on the results of our study, the precise phenotypic characterization of patients and families should support molecular testing and could advance the management of diagnosis, genetic counseling, and selection procedures for randomized clinical trials.

## Notes

Supplement. **eTable 1. **Distribution of Clinical Categories of Relatives Associated With Clinical Categories of Probands **eTable 2. **Clinical Summary of Probands and Relatives by Category **eFigure 1. **Population of the Italian Clinical Registry for FSHD **eFigure 2. **Distribution of Clinical Phenotypes in Families
