# Supplementary material for: Phenotypic Variability Among Patients With D4Z4 Reduced Allele Facioscapulohumeral Muscular Dystrophy
Source: JAMA Netw Open. 2020 May 1;3(5):e204040. doi: 10.1001/jamanetworkopen.2020.4040 (PMC7195625; doi:10.1001/jamanetworkopen.2020.4040)
Supplement: Supplement. — eTable 1. Distribution of Clinical Categories of Relatives Associated With Clinical Categories of Probands eTable 2. Clinical Summary of Probands and Relatives by Category eFigure 1. Population of the Italian Clinical Registry for FSHD eFigure 2. Distribution of Clinical Phenotypes in Families [file jamanetwopen-3-e204040-s001.pdf]

Supplementary Online Content

Ruggiero L, Mele F, Mangenelli F, et al. Phenotypic variability among patients with D4Z4 reduced allele facioscapulohumeral muscular dystrophy. *JAMA Netw Open*. 2020;3(5):e204040. doi:10.1001/jamanetworkopen.2020.4040

eTable 1. Distribution of Clinical Categories of Relatives Associated With Clinical Categories of Probands
eTable 2. Clinical Summary of Probands and Relatives by Category
eFigure 1. Population of the Italian Clinical Registry for FSHD
eFigure 2. Distribution of Clinical Phenotypes in Families

This supplementary material has been provided by the authors to give readers additional information about their work.

© 2020 Ruggiero L et al. *JAMA Network Open.*

eTable 1. Distribution of Clinical Categories of Relatives Associated With Clinical Categories of Probands

CCEF categories				
Relatives	*Probands*			
	**A** (n 62)	**B** (n 19)	**C** (n 1)	**D** (n 24)
**A** (n (%))	30 (22.4%)	2 (6.1%)	0	5 (8.5%)
**B** (n (%))	31 (23.1%)	10 (30.3%)	0	9 (15.2%)
**C** (n (%))	62 (46.3%)	16 (48.5%)	1 (100%)	40 (67.8%)
**D** (n (%))	11 (8.2%)	5 (15.1%)	0	5 (8.5%)

© 2020 Ruggiero L et al. *JAMA Network Open.*

eTable 2. Clinical Summary of Probands and Relatives by Category

Characteristic	Category A	Category B	Category C	Category D	ANOVA *P* value
**Probands**					
No. (men/women)	99 (59/40)	36 (25/11)	2 (2/0)	50 (17/33)	NA
Age at evaluation, mean (SD), y	52.2 (15.1)[a]	50.1 (15.7)[a]	40.5 (14.8)	59.0 (13.7)	.014
Age at onset, mean (SD), y	29.1 (16.4)[a]	35.1 (18.4)	NA[b]	40.9 (17.8)	<.001
FSHD score, mean (SD)	6.7 (3.3)[c]	3.1 (1.6)	NA[b]	6.4 (3.4)[c]	<.001
**Relatives**					
No. (men/women)	38 (20/18)	51 (23/28)	124 (56/68)	22 (5/17)	NA
Age at evaluation, mean (SD), y	51.6 (19.1)[d]	43.7 (14.1)	40.8 (15.3)	60.9 (17.0)[d]	.004
Age at onset, mean (SD), y	29.2 (17.6)[a]	32.6 (13.3)	–[b]	43.6 (18.0)	.016
FSHD score, mean (SD)	4.8 (2.8)	1.8 (1.4)[e]	–[b]	5.1 (4.2)[e]	<.001

Abbreviations: ANOVA, analysis of variance; FSHD, facioscapulohumeral muscular dystrophy, NA, not applicable.

[a]Significantly different from category D.

[b]Indicates an asymptomatic participant; therefore, age at onset and FSHD score are not present

[c]Significantly different from category B.

[d]Significantly different from category C.

[e]Significantly different from category A.

© 2020 Ruggiero L et al. *JAMA Network Open.*

eFigure 1. Population of the Italian Clinical Registry for FSHD

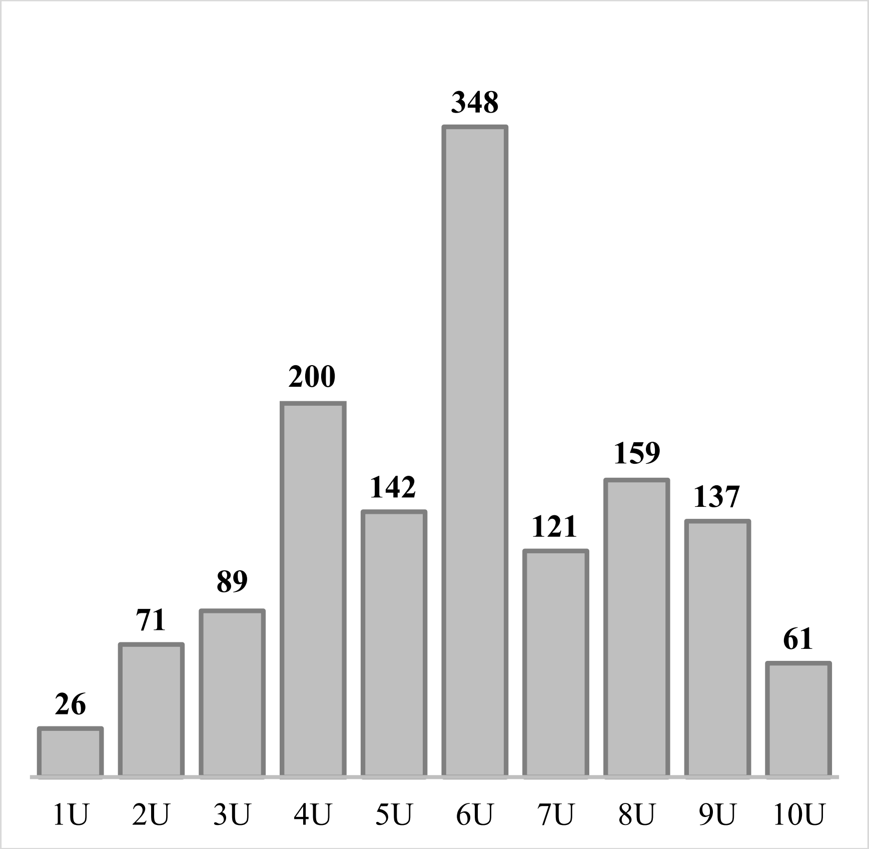

eFigure1 Frequency of alleles in INRF

Allele size distribution in the group of probands in Italian national registry for FSHD

© 2020 Ruggiero L et al. *JAMA Network Open.*

eFigure 2. Distribution of Clinical Phenotypes in Families

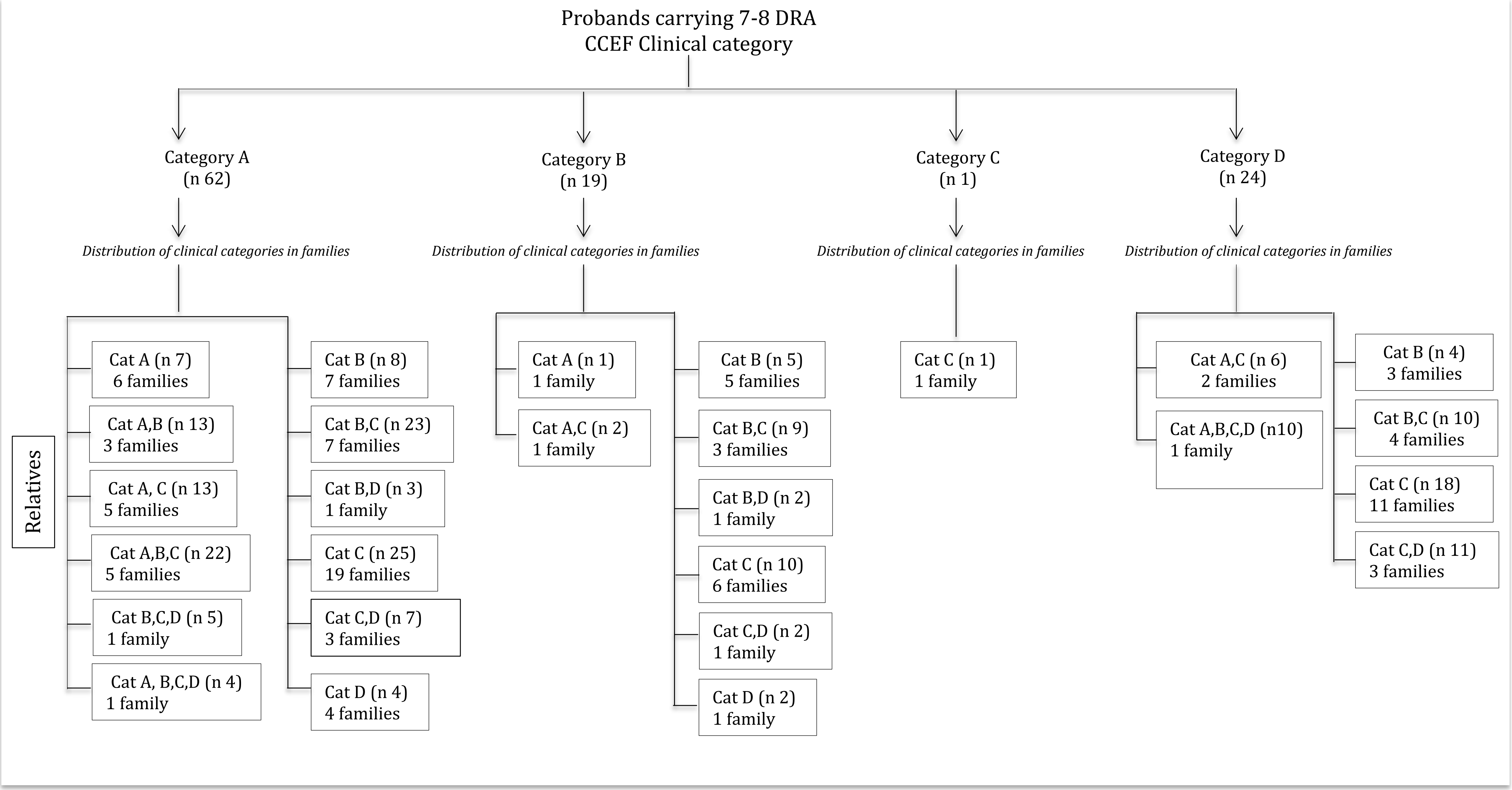

© 2020 Ruggiero L et al. *JAMA Network Open.*

eFigure2 Distribution of clinical phenotypes in families.

The clinical patterns observed in families in which one 7-8 DRA segregates is described. Families were grouped on the basis of the clinical category of the probands and sub-grouped on the basis of the clinical patterns assessed in relatives. Only probands with at least one family member available for the analysis (106 out of 187) are included

© 2020 Ruggiero L et al. *JAMA Network Open.*
